# Supplementary material for: Pre-complexation of talin and vinculin without tension is required for efficient nascent adhesion maturation
Source: eLife. 2021 Mar 30;10:e66151. doi: 10.7554/eLife.66151 (PMC8009661; doi:10.7554/eLife.66151)
Supplement: Supplementary file 1. — (A) Features used for classification of adhesions. (B) Nine adhesion groups defined heuristically. (C) Automatic labeling criteria. [file elife-66151-supp1.docx]

Supplementary Table 1A Features used for classification of adhesions

|  | Feature name | Definition | Targets | Range | Unit |
| --- | --- | --- | --- | --- | --- |
| 1 | Max Intensity | Maximum intensity over adhesion lifetime, normalized by the max intensity | high for G2, low for G1 | 0-1 | 1 |
| 2 | Mean intensity | Mean intensity over adhesion lifetime, normalized by the max intensity | high for G2, G6, G7 and G8, low for G1, G3, G4 and G5 | 0-1 | 1 |
| 3 | Initial intensity | Fluorescent intensity of the track at the start of the track, normalized by the max intensity | High for G7, low for both G1 and G2 | 0-1 | 1 |
| 4 | Relative intensity decay | (Max intensity – intensity at the end of the track) / Max intensity | G1: big G2: small | 0-1 | 1 |
| 5 | Lifetime | Adhesion lifetime | G9: short  G2, G7, G8: long | 1-600 | Sec |
| 6 | Rise time | Time from adhesion initiation to time of max intensity  Time at max intensity – time at the beginning of the track | High for G2 | 1-600 | Sec |
| 7 | Overall intensity slope | Slope of intensity over entire adhesion life time | Positive for G1 and G2 |  | a.u./sec |
| 8 | Early intensity slope | Slope of intensity during initial one minute | Positive for G1 and G2 |  | a.u./sec |
| 9 | Late intensity slope | Slope of intensity during late one minute | G1 < G2 | -1 to 1 | a.u./sec |
| 10 | Initial adhesion motion | Adhesion motion in the direction of protrusion for the first minute of lifetime. | Positive for G3 and G4 |  | µm |
| 11 | Terminal adhesion motion | Adhesion motion in the direction of protrusion for the last minute of lifetime. | Negative for G4 and G6 |  | µm |
| 12 | Max edge fluctuation | Maximum fluctuation of local edge protrusion during adhesion lifetime | High for G1, G2, G3 |  | µm |
| 13 | Distance to edge at initiation | Closest distance of adhesion to the cell edge at initiation | Low for G1, G2, G3, G4, G7, high for G5 and G8 |  | µm |
| 14 | Distance to edge at termination | Closest distance of adhesion to the cell edge at termination of adhesion’s life | Low for G3 and G4, medium for G1 and G2, high for G5 and G8 |  | µm |
| 15 | Change in distance to edge during lifetime | Difference in closest distance of adhesion to the cell edge for entire adhesion lifetime | Low for G7, negative for G6 |  | µm |
| 16 | Variance of edge fluctuation | Standard deviation of edge fluctuation of local edge protrusion during adhesion lifetime | Zero for G9 (adhesions near image boundary) |  | µm |
| 17 | Focal adhesion area | Segmented area for focal complexes (>0.5 µm in length) and focal adhesions (>2 µm in length) | Positive for G2 and G8 | 0.1 to >10 | µm^2^ |
| 18 | Adhesion status at termination | The time of the last ‘FA’ status compared to the time at termination of adhesion lifetime, normalized with the adhesion lifetime. E.g. 0 when the adhesion is ending as a FA status at the end of the movie. | Near zero for G2 and G8. Intermediate value (e.g. 0.3-0.7) for G1, G3 and G4. | 0 to 1 | 1 |
| 19 | Time period as NA before FC or FA | Time period of the adhesion as ‘NA’ status before the first ‘FC’ or ‘FA’ status. | Near zero for G7 and G8. At least 20 seconds for G2. | 0 to the time of a movie | sec |
| 20 | Edge protrusion speed | An average speed of the edge closest to the adhesion in the projected direction outward normal to the edge for adhesion lifetime. | Positive for G1, G2, G3, negative for G6 | -1 to 1 | µm/min |
| 21 | Adhesion movement speed | A speed of the adhesion in the projected direction outward normal to the edge for adhesion lifetime. | positive for G3, slightly negative for G1 and G2, highly negative for G6 | -1 to 1 | µm/min |
| 22 | Texture homogeneity | Out of area of 10x10 pixel around x-y position, gray-level co-occurrence matrix is calculated with an offset of (0,1), then homogeneity property is calculated as  $\sum_{i,j} \frac{p\left( i,j \right)}{1+\left\vert i-j \right\vert}$  where p is the co-occurrence matrix, i and j are row and column indices, respectively. | High for true focal adhesions G2 and G8, low for nascent adhesions (G1, G3, G4) even when the intensity is high. | 0 to 1 | 1 |

Supplementary Table 1B. Nine adhesion groups that are defined heuristically.

| Class | Short name | Qualitative description |
| --- | --- | --- |
| G1 | NAs turn-over | NAs that form at the edge but stays at their positions or slide rearward as the edge protrude forward and go on turn-over. |
| G2 | NAs maturing | NAs that form at the edge but stays at their positions or slide rearward as the edge protrude forward and *mature* into FCs and FAs |
| G3 | NAs moving along protruding edge | NAs that form at the edge and move forward with protruding edge. |
| G4 | NAs at stalling edge | NAs that form at an initially protruding edge and stay at the edge which becomes stalling. |
| G5 | NAs inside | NAs with low fluorescence intensity at the cell interior |
| G6 | FAs retracting | FAs or FCs at the retracting edge |
| G7 | FAs stable at the edge | Stable adhesions (FAs) at the static cell edge |
| G8 | FAs stable inside | FAs with high fluorescence intensity and long lifetime at the cell interior |
| G9 | noise or very transient | Insignificant tracks with short life time (< 5 sec), low intensity or large random movement |

Supplementary Table 1C. Automatic labeling criteria. The sign ‘-‘ means that the corresponding criteria was not used for automatic labeling.

|  | Adhesion Classes | G1 | G2 | G3 | G4 | G5 | G6 | G7 | G8 | G9 |
| --- | --- | --- | --- | --- | --- | --- | --- | --- | --- | --- |
| Automatic labeling criteria | Edge velocity | Positive | Positive | Positive | Negative | Small | - | Positive | - |  |
|  | Relative distance to edge | Increasing | Increasing | Constantly small | - | Constantly small | Large | - | Large | Large |
|  | Starting location | At the edge | At the edge | At the edge | - | - | - | At the edge | - | - |
|  | Initial trend of amplitude | Rising | Rising | - | - | - | - | - | - | - |
|  | Ending trend of amplitude | Decaying | Non-decaying | - | - | - | - | - | - | - |
|  | Relative time of maximum intensity | Early in the lifetime | Late in the lifetime | - | - | - | - | - | - | - |
|  | Overlapping with FA segmentation | Yes, but only up to small area | Yes, especially at the end | No | - | - | - | - | - | - |
|  | Mean amplitude | High enough | High enough | - | - | High | Low | Low | High | Low |
|  | Initial amplitude | Low enough | Low enough | - | - | - | - | - | - | - |
|  | Lifetime with ‘NA’ status (not overlapping with segmentation) | At least 10 sec | At least 10 sec | - | - | - | - | - | - | - |
|  | Image texture homogeneity | - | High | - | - | - | Low | - | - | - |
|  | Adhesion velocity | - | - | Positive | Negative | - | - | - | - | - |
|  | Slope of amplitude | - | - | - | Negative | - | - | - | - | - |
|  | Lifetime | - | - | - | - | Long | Short | - | Long | At least twice of G6 |
|  | Edge velocity at last 2 minutes of an adhesion’s lifetime | - | - | Positive | - | - | - | Near zero | - | - |
